# Pharmacological effects of *Salvia miltiorrhiza-*derived interventions on osteoporosis in animal models: a systematic review and meta-analysis

**DOI:** 10.3389/fphar.2026.1740974

**Published:** 2026-05-26

**Authors:** Yuanna Zhang, Rui Tang, Dongping Wan, Haodong Wu, Xiang Ji, Rui Wang, Chuan Leng, Shihang Cao, Xi Gao

**Affiliations:** 1 Honghui Hospital, Xi’an Jiaotong University, Xi’an, Shanxi, China; 2 The Clinical Medical College, Chengdu University of Chinese Traditional Medicine, Chengdu, Sichuan, China; 3 The First Clinical Medical College, Guangxi University of Chinese Medicine, Nanning, Guangxi, China

**Keywords:** animal models, bone metabolism, bone mineral density, osteoporosis, *Salvia miltiorrhiza*, systematic review

## Abstract

**Purpose:**

This systematic review and meta-analysis aimed to evaluate the pharmacological effects of *Salvia miltiorrhiza*-derived interventions, including extracts and bioactive metabolites, in treating osteoporosis in animal models.

**Methods:**

A comprehensive literature search was conducted across seven Chinese and English databases. Studies included in the analysis were randomized controlled trials (RCTs) assessing the effects of *Salvia miltiorrhiza*-derived extracts or bioactive metabolites on osteoporosis in animal models. Data from 24 eligible studies were extracted, including information on bone density, bone morphology, and biochemical markers. The risk of bias was assessed using the SYRCLE’s risk of bias tool, and statistical analyses were performed using Stata and Review Manager.

**Results:**

*Salvia miltiorrhiza*-derived interventions significantly improved Bone Mineral Density (BMD) (SMD = 1.95, 95% CI = 1.48 to 2.42, p < 0.000001), trabecular structure, and biomechanical properties. *Salvia miltiorrhiza*-derived interventions also modulated key bone metabolism markers, including increased procollagen type I N-terminal propeptide (PINP) and decreased tartrate-resistant acid phosphatase (TRACP) and alkaline phosphatase (ALP) levels, indicating improved osteogenesis and reduced bone resorption. Subgroup analyses revealed that the ovariectomy (OVX) model showed the most significant effects, with more favorable outcomes in the higher-dose subgroup and in studies with intervention durations of less than 12 weeks.

**Conclusion:**

*Salvia miltiorrhiza*-derived interventions demonstrated significant bone-protective effects in osteoporosis animal models. However, these findings should be interpreted within the context of preclinical evidence, as animal models have limited translational validity and do not fully reflect human pharmacokinetics or disease complexity. Further well-designed preclinical and translational studies are needed before any implications for human application can be considered.

## Introduction

Osteoporosis (OP) is a prevalent metabolic bone disorder characterized by reduced bone mass, microarchitectural deterioration, and decreased bone strength, leading to an increased risk of fractures (Liu et al., 2022; Morin et al., 2025) This condition is commonly linked to factors such as age, gender, genetic predispositions, lifestyle choices, and certain chronic diseases (Jiang S. et al., 2025). As the global population continues to age, the incidence of OP has been rising annually (Zhao Y. et al., 2025). In Europe, North America, and other regions, approximately half of elderly women are affected by OP, with a significant prevalence in men as well (Kanis et al., 2021). The clinical manifestation of OP typically begins with fractures, especially in the hip, spine, and wrist. These fractures can have serious consequences, often leading to functional impairments and reduced quality of life. The pathogenesis of OP is complex and involves an imbalance in bone metabolism, primarily characterized by excessive bone resorption and insufficient bone formation (Walker and Shane, 2023). Recent research has provided further insight into the molecular mechanisms associated with OP, including oxidative stress, inflammatory responses, and the interactions between osteoblasts and osteoclasts (Luo et al., 2025; Zhao H. et al., 2025). Moreover, the relationship between OP and other chronic diseases, such as diabetes, chronic kidney disease, and cardiovascular diseases, has received significant attention (Zhang et al., 2025; Kang et al., 2025; Rodríguez-Gómez et al., 2022). Despite the availability of various treatments, such as anti-resorptive drugs (e.g., bisphosphonates) and bone-forming agents (e.g., recombinant human parathyroid hormone (PTH)), significant challenges persist due to the heterogeneity of OP and individual variations in treatment response (Noh et al., 2020; Yamamoto et al., 2024). Therefore, there is an urgent need for novel therapeutic strategies to effectively address this global health issue (Kim et al., 2021).

In recent years, natural products have garnered widespread attention due to their therapeutic potential in treating various diseases. *Salvia miltiorrhiza* Bunge (Lamiaceae), commonly known as Danshen, a botanical drug source, is widely used in East Asia (Lee et al., 2024). Traditionally, *S. miltiorrhiza* has been used to treat cardiovascular diseases, blood stasis, pain, and inflammation (Lee et al., 2024; Lan et al., 2025; Li P. et al., 2025). Recent studies have expanded the therapeutic applications of *S. miltiorrhiza*, particularly in bone health, highlighting its potential in treating OP (Wang et al., 2024). *Salvia miltiorrhiza*-derived interventions contain a variety of bioactive metabolites, including tanshinones and phenolic acids, which demonstrate significant efficacy in bone homeostasis (Khan et al., 2025). Current pharmacological research indicates that *S. miltiorrhiza* exhibits various biological activities, including anti-inflammatory, antioxidant, and bone-protective effects (Wang et al., 2025; Deng et al., 2024). Studies suggest that *S. miltiorrhiza* could modulate the activity of osteoblasts and osteoclasts by regulating the bone remodeling process, thereby improving bone density and reducing bone resorption (Wang et al., 2022).

However, despite promising results from preclinical studies of *S. miltiorrhiza*, inconsistencies across experimental findings highlight the need for systematic evidence synthesis and quantitative evaluation. Meta-analysis, as a valuable method for integrating data from multiple studies, helps reduce bias and improves the reliability of research conclusions (Xu et al., 2025). To date, previous evidence syntheses have either focused on single constituents or provided qualitative summaries, rather than quantitatively comparing the anti-osteoporotic effects of different *S. miltiorrhiza*-derived extracts and bioactive metabolites across multiple animal models (Wang et al., 2022). To address this gap, this study aimed to evaluate the protective effects of different *S. miltiorrhiza*-derived extracts and bioactive metabolites in OP animal models. By integrating data from multiple animal studies, this research provided a broader quantitative assessment of efficacy and further explored variations related to extract type, experimental model, and treatment characteristics. Accordingly, this study was designed to provide analytical insight beyond a simple confirmatory update of the existing literature and to offer a basis for future preclinical and translational research rather than direct clinical extrapolation.

## Methods

We conducted this meta-analysis in full compliance with the Preferred Reporting Items for Systematic Reviews and Meta-Analyses (PRISMA) standards for systematic reviews and meta-analyses (Liberati et al., 2009) and it was officially registered with PROSPERO under the ID CRD420251151733.

### Literature search strategy

We conducted a literature search in seven Chinese and English databases, including Web of Science, PubMed, Scopus, Embase, Foreign Medical Literature Retrieval Service (FMRS), China National Knowledge Infrastructure (CNKI), and Wanfang Data Knowledge Service Platform. The literature screening process was independently carried out by two authors, using a search strategy that combined keywords with Medical Subject Headings (MeSH). The specific combinations of search terms (as detailed in Supplementary Table S1) were used for the inclusion of studies from the inception of each database until 1 October 2025.

### Taxonomic validation of the botanical drug

The botanical drug source investigated in this review was taxonomically validated as *S. miltiorrhiza* Bunge (Lamiaceae), commonly known as Danshen. Because the present study was a systematic review rather than an original pharmacognostic investigation, taxonomic authentication and voucher-specimen information could only be assessed from the original publications when reported. Such information was not consistently available across the included studies and should therefore be considered when interpreting the evidence.

### Inclusion and exclusion criteria for literature screening

This systematic review and meta-analysis included randomized controlled animal studies that compared *S. miltiorrhiza*-derived interventions with saline or placebo in OP animal models. The inclusion criteria were as follows: a) Subjects were rats or mice with successfully established OP models; b) The study type was *in vivo*; c) The outcome measures were clearly defined and the data were extractable for analysis; d) The study design was a randomized controlled trial. The exclusion criteria were: a) Studies involving animal models of other bone metabolic diseases; b) *In vitro* studies or those involving combined drug regimens or compound formulations; c) Studies with data previously published; d) Non-original research, such as conference abstracts, literature reviews, expert comments, or letters to the editor. Because the included interventions were all derived from *S. miltiorrhiza* but represented different phytochemical classes, the primary meta-analysis was intended to provide a class-level estimate of anti-osteoporotic efficacy, while heterogeneity related to intervention composition was further explored through subgroup analysis by phytochemical class.

### Data extraction

After removing duplicate studies, two researchers independently and in a double-blind manner screened the titles and abstracts of the remaining studies and excluded those that did not meet the predefined inclusion criteria. For the studies that passed the preliminary screening, full-text reviews were conducted to confirm their eligibility for inclusion. In cases of disagreement during the screening process, the researchers reached consensus through discussion or by arbitration from a third researcher. Data extraction was independently carried out by two researchers in strict accordance with the double-blind principle. The extracted data included: first author information, publication year, methods for establishing the OP model, animal weight and age, sample size, intervention methods and administration routes, experimental duration (with specified time units), and the mean and standard deviation (SD) of primary efficacy indicators. For graphically presented data, the researchers used GetData Graph Digitizer Software (Version 2.26) for visual extraction and numerical reconstruction.

### Quality assessment

This study used the SYRCLE’s risk of bias tool to independently assess the risk of bias in the included studies (Hooijmans et al., 2014). The assessment included six areas of bias: selection bias, performance bias, detection bias, attrition bias, reporting bias, and other biases, with a total of ten items. If a study satisfies the criteria in all areas, it is classified as having a low risk of bias; if it fails to meet the criteria, it is classified as having a high risk of bias. If there is insufficient information to make a clear judgment, the risk of bias is labeled as uncertain. During the evaluation process, if disagreements arise between reviewers, they were resolved through discussion and consultation to guarantee the accuracy and consistency of the findings.

### Outcome measures

The primary outcome measure of this study is bone mineral density (BMD). The secondary outcome measures include bone morphological parameters (trabecular number, trabecular thickness, trabecular separation, bone volume fraction), biomechanical properties of bone (maximum load, ultimate stress, elastic modulus, structural model index), and bone biochemical markers (procollagen type I N-terminal propeptide (PINP), estradiol (E2), serum alkaline phosphatase (ALP), serum osteocalcin (OC), tartrate-resistant acid phosphatase (TRACP), serum calcium, serum phosphorus).

### Statistical analysis

Data analysis was conducted using Stata software (Stata SE, version 18) and Review Manager 5.4.0 for data processing and visualization. Heterogeneity was assessed using the I^2^ statistic, which quantifies heterogeneity. A fixed-effects model was used when the I^2^ value was less than 50%. If the I^2^ value was greater than or equal to 50%, a random-effects or fixed-effects model was selected depending on the source of heterogeneity. To investigate the sources of heterogeneity in more detail, subgroup analysis and sensitivity analysis were conducted. Sensitivity analysis was performed using the leave-one-out approach to verify the robustness of the results. For continuous data, mean differences (MDs) or standardized mean differences (SMD) with 95% confidence intervals (CI) were calculated, with a significance level of p < 0.05. To further assess whether the pooled effect size might have been influenced by methodological bias or small-study effects, additional analyses were performed. These included sensitivity analysis after exclusion of studies judged to be at high risk of bias, visual inspection of funnel plot asymmetry, Egger’s regression test, and trim-and-fill analysis.

## Results

### Search results

**FIGURE 1:**
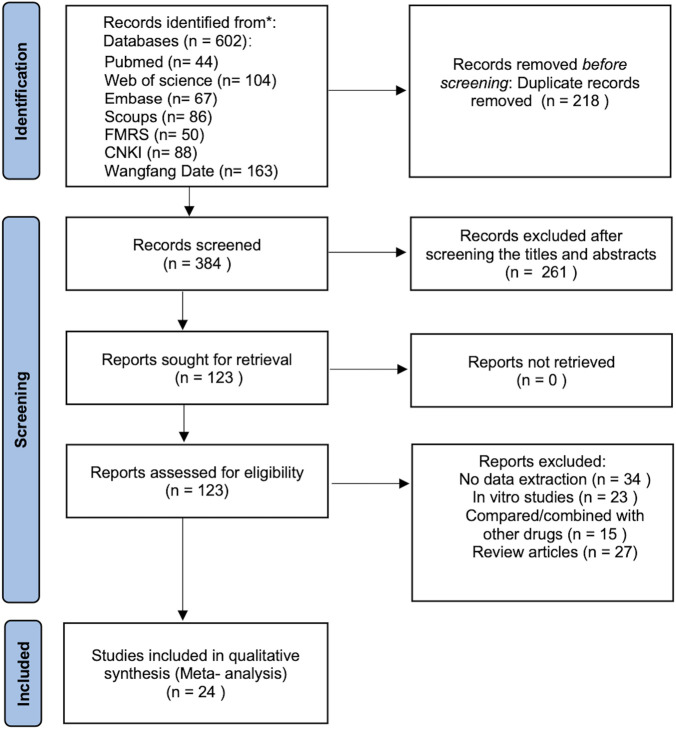
PRISMA flow chart of study selection.

The literature inclusion process of this study is illustrated in Figure 1. After searching seven databases in both Chinese and English, a total of 602 studies were initially selected, of which 218 were excluded due to duplication. After reviewing the titles and abstracts of the remaining studies, 261 studies were excluded because they were irrelevant. We then retrieved the full texts of the remaining 123 studies and carefully reviewed them. In the end, 99 studies were excluded for the following reasons: a) 34 studies could not provide data on the primary outcome measures; b) 15 studies involved comparisons or combined use with other drugs; c) 23 studies were *in vitro* experiments; d) 27 studies were review articles. Ultimately, 24 studies were included in the analysis, consisting of 12 Chinese studies (Yuan, 2013; Miao et al., 2012; Ma and Han, 2022; Chen et al., 2015; Xu et al., 2010; Peng et al., 2021; Qu et al., 2016; ChenJ et al., 2015; Wang X. et al., 2019; Yang et al., 2018; Dong et al., 2021; Yu et al., 2012) and 12 English studies (Panwar et al., 2017; Lee et al., 2020; Cui et al., 2011; Yang et al., 2022; Park et al., 2017; Liu et al., 2018; Qiu et al., 2025; Liuyuan and Shen, 2018; Cui et al., 2012; Lai et al., 2021; Chen et al., 2017).

### Literature characteristics

These 24 studies were published between 2010 and 2025, with details summarized in Table 1. Of these, one study used retinoic acid for gavage induction, two studies used a diabetes-induced model, two studies used a disuse-induced OP model, six studies used glucocorticoid induction, and the remaining 13 studies used ovariectomy (OVX) to induce the OP model. Regarding the major bioactive metabolite classes represented in *S. miltiorrhiza*, all studies reported on this. Specifically, four studies used ethanol extraction, three studies used the decoction method, five studies used tanshinone as the active ingredient, and the remaining 12 studies used salvianolic acid as the active substance. 23 studies reported the administration route as oral gavage, one study used intraperitoneal injection, with doses ranging from 5 mg/kg/day to 15 g/kg/day, and the duration of the experiments ranged from 4 weeks to 16 weeks.

**TABLE 1:** Characteristics of the included studies.

First author	Induction of osteoporosis	Species	Gender	Effective substance	Sample size		Intervention		Methods of administration	Duration of study
					IG	CG	IG	CG		
Yuan (2013)	DOM	Wistar	Male	AESM	12	12	15 g/kg/day	PS	Intragastric	4 weeks
Miao et al. (2012)	Diabetic	Wistar	Male	AESM	10	10	5 g/kg/day	PS	Intragastric	8 weeks
Ma and Han (2022)	OVX	SD	Female	Salvianolic acid ester	10	10	40 mg/kg/day	PS	Intraperitoneal injection	4 weeks
Chen et al. (2015)	Glu	SD	Female	Salvianolic acid B	10	9	25 mg/kg/day	PS	Intragastric	14 weeks
Xu et al. (2010)	RA	SD	Female	Danshensu	8	8	5 mg/kg/day	PS	Intragastric	4 weeks
Peng et al. (2021)	OVX	SD	Female	Danshensu	12	12	40 mg/kg/day	PS	Intragastric	4 weeks
Qu et al. (2016)	OVX	SD	Female	Danshensu	10	10	12.5 mg/kg/day	DW	Intragastric	12 weeks
ChenJ et al. (2015)	Glu	SD	Female	Danshensu	10	10	25 mg/kg/day	PS	Intragastric	14 weeks
Wang et al. (2019a)	DOM	Wistar	Female	Tanshinone IIA	10	10	11 mg/kg/day	DW	Intragastric	4 weeks
Yang et al. (2018)	OVX	SD	Female	Tanshinone IIA	10	10	11 mg/kg/day	DW	Intragastric	12 weeks
Wang et al. (2021)	OVX	SD	Female	Salvianolic acid B	12	12	25 mg/kg/day	PS	Intragastric	12 weeks
Yu et al. (2012)	Glu	SD	Male	AESM	8	8	375 mg/kg/day	PS	Intragastric	12 weeks
Panwar et al. (2017)	OVX	C57BL/6	Female	Tanshinone IIA	10	9	40 mg/kg/day	Vehicle	Intragastric	12 weeks
Lee et al. (2020)	OVX	ICR	Female	EESM	6	6	50 mg/kg/day	PS	Intragastric	12 weeks
Cui et al. (2011)	OVX	SD	Female	EESM	9	10	30 mg/kg/day	Vehicle	Intragastric	8 weeks
Yang et al. (2022)	OVX	SD	Female	Cryptotanshinone	8	8	20 mg/kg/day	PS	Intragastric	4 weeks
Park et al. (2017)	OVX	ICR	Female	EESM	7	7	100 mg/kg/day	PS	Intragastric	12 weeks
Liu et al. (2018)	OVX	SD	Female	EESM	9	9	5 g/kg/day	DW	Intragastric	14 weeks
Qiu et al. (2025)	OVX	C57BL/6	Female	Salvianolic acid A	6	6	5 mg/kg/day	PS	Intragastric	12 weeks
Liuyuan et al. (2018)	OVX	SD	Female	Salvianolic acid B	10	10	40 mg/kg/day	PS	Intragastric	12 weeks
Cui et al. (2012)	Glu	SD	Male	Salvianolic acid B	8	8	80 mg/kg/day	PS	Intragastric	12 weeks
Lai et al. (2021)	Glu	SD	Male	Danshensu	12	12	25 mg/kg/day	PS	Intragastric	16 weeks
Chen et al. (2017)	Glu	SD	Female	Danshensu	16	24	25 mg/kg/day	PS	Intragastric	14 weeks
Jiang et al. (2025a)	Diabetic	C57BL/6	Male	Tanshinone IIA	10	10	30 mg/kg/day	PS	Intragastric	6 weeks

OVX, ovariectomy; Glu, Glucocorticoid; DOM, disuse osteoporosis model; RA, retinoic acid; ICR, institute of cancer research; AESM, aqueous extract of salvia miltiorrhiza; EESM, ethanolic extract of salvia miltiorrhiza; IG, intervention group; CG, control group; PS, physiological saline; DW, distilled water.

### Quality appraisal of the literature

**FIGURE 2:**
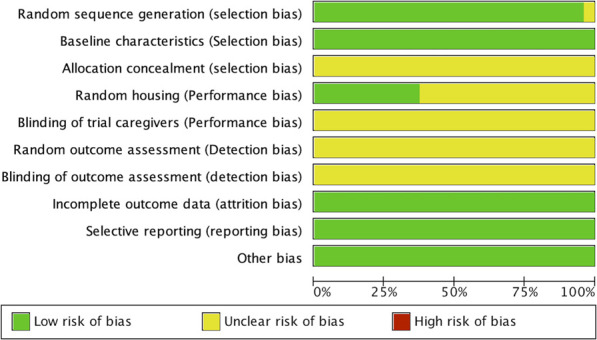
Risk-of-bias assessment of the included animal studies using SYRCLE’s risk of bias tools.

The risk of bias in the animal studies was assessed using the SYRCLE’s risk of bias tool, with the results presented in Figure 2. During the evaluation, it was found that one study did not mention random sequence generation, only nine studies reported random housing, and none of the studies reported allocation concealment, blinding of experimenters, or randomization and blinding of outcome assessment. All included studies reported baseline data and had no issues with incomplete outcome data, selective reporting, or other risks of bias.

### Bone mineral density and subgroup analyses

**FIGURE 3:**
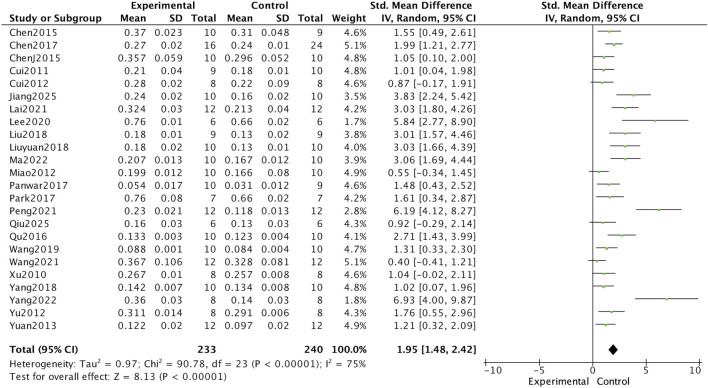
Forest plot comparing BMD between the *Salvia miltiorrhiza*-derived interventions group and the control group.

This study included 24 experimental datasets for analysis, confirming the efficacy of *S. miltiorrhiza*-derived interventions in improving BMD in rodent OP models. Figure 3 shows that the bone mineral density in the *S. miltiorrhiza*-derived interventions treatment group was significantly higher than in the control group (SMD = 1.95, 95% CI = 1.48 to 2.42, p < 0.000001). Additionally, as shown in Table 2, subgroup analysis of BMD was conducted based on model type, species, phytochemical class of intervention, dose, and intervention duration to further elucidate the sources of heterogeneity and determine the optimal treatment strategy. Variation in the I^2^ values across different models, species, and phytochemical classes suggests that these factors may contribute to heterogeneity in BMD outcomes. The phytochemical-class subgroup analysis showed positive pooled effects across aqueous extract, ethanolic extract, tanshinone-class, and salvianolic acid-class interventions, although the magnitude of effect differed among subgroups. Method-based subgroup analysis showed significant pooled effects in studies using DEXA (SMD = 1.95, 95% CI = 1.31–2.60), Micro-CT (SMD = 2.80, 95% CI = 1.66–3.95), and other methods (SMD = 1.24, 95% CI = 0.61–1.87). Site-based subgroup analysis also showed significant pooled effects for femoral measurements (SMD = 2.29, 95% CI = 1.70–2.87) and other skeletal sites (SMD = 1.15, 95% CI = 0.63–1.67). Other categories showed little variation in I^2^ values during subgroup analysis, indicating that these factors are likely not sources of heterogeneity. In the subgroup analysis, studies using doses above 30 mg/kg/day showed a numerically larger pooled effect on BMD than those using doses of 30 mg/kg/day or less; however, this finding should be interpreted cautiously because dose ranges differed markedly across phytochemical classes and were not directly comparable. Similarly, studies conducted in Sprague-Dawley (SD) rats, OVX models, and those involving tanshinone-class interventions or intervention durations of less than 12 weeks tended to show relatively larger pooled effects.

**TABLE 2:** Subgroup analysis of bone mineral density based on multiple factors related to Salvia miltiorrhiza treatment.

Subgroup	Standardized mean difference (95% confidence interval)	I^2^	p value
Species			
SD	2.10 [1.48, 2.71]	78	0.000
Wistar	1.01 [0.48, 1.54]	0	0.000
C57	1.98 [0.47, 3.50]	77	0.000
ICR	3.48 [-0.64, 7.60]	84	0.10
Model			
OVX	2.48 [1.63, 3.33]	82	0.000
GLU	1.67 [1.09, 2.24]	47	0.000
DOM	1.25 [0.60, 1.91]	0	0.000
Diabetic	2.12 [-1.09, 5.33]	92	0.19
Effective substance			
AESM	1.09 [0.45, 1.73]	24	0.000
EESM	2.39 [0.92, 3.85]	75	0.000
Tanshinone	2.42 [1.08, 3.75]	82	0.000
Salvianolic acid	1.97 [1.28, 2.66]	78	0.000
Dose			
≤30 mg/kg/day	1.73 [1.15, 2.31]	73	0.000
>30 mg/kg/day	2.27 [1.45, 3.08]	78	0.000
Duration of intervention			
<12 weeks	2.40 [1.36, 3.43]	85	0.000
≥12 weeks	1.75 [1.27, 2.24]	64	0.000
Measurement method			
DXA	1.95 [1.31, 2.60]	79	0.000
Micro-CT	2.80 [1.66, 3.95]	81	0.000
Other method	1.24 [0.61, 1.87]	0	0.000
Measurement site			
Femur	2.29 [1.70, 2.87]	79	0.000
Other skeletal site	1.15 [0.63, 1.67]	4	0.000

### Skeletal histomorphometry

**FIGURE 4:**
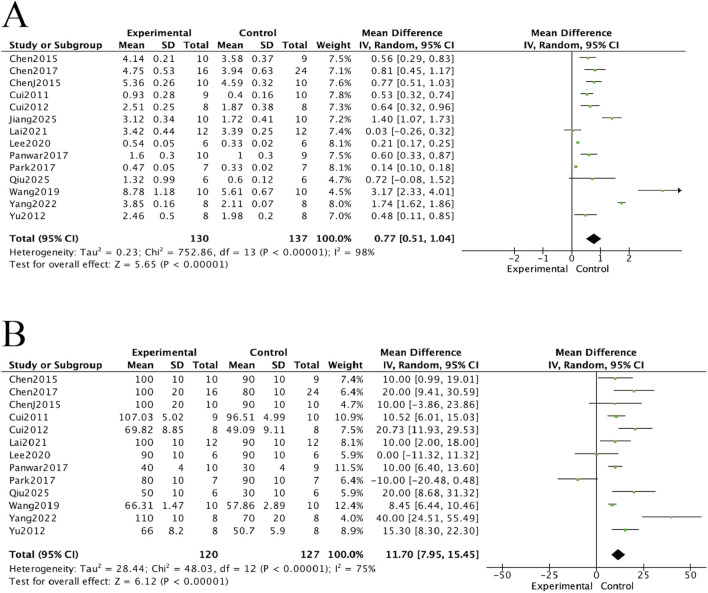
Forest plots of the effects of *Salvia miltiorrhiza*-derived interventions on trabecular microarchitecture in osteoporosis animal models. **(A)** Trabecular number. **(B)** Trabecular thickness.

**FIGURE 5:**
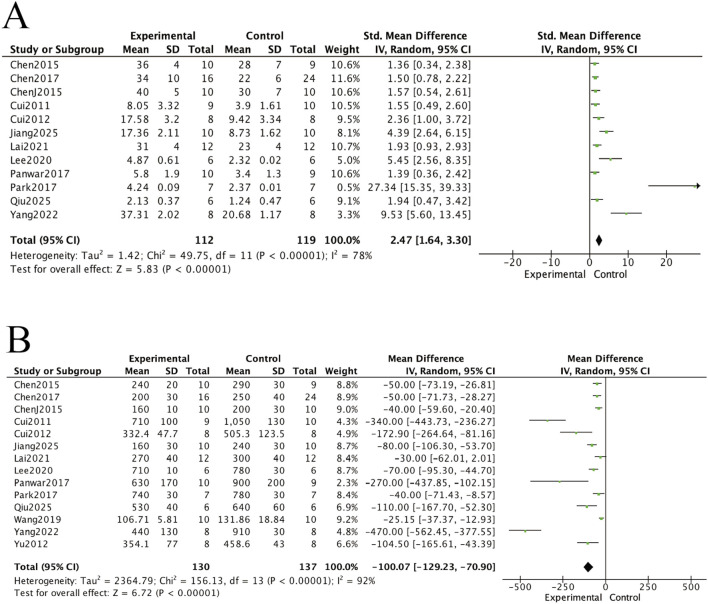
Forest plots of the effects of *Salvia miltiorrhiza*-derived interventions on bone volume fraction and trabecular separation in osteoporosis animal models. **(A)** Bone volume fraction. **(B)** Trabecular separation.

A meta-analysis of the effects of *S. miltiorrhiza*-derived interventions on bone tissue morphology in OP animal models is presented in Figures 4, 5. Figure 4 shows that 14 studies reported the impact of *S. miltiorrhiza*-derived interventions on trabecular number (MD = 0.77, 95% CI = 0.51 to 1.04, p < 0.000001). Thirteen studies demonstrated that *S. miltiorrhiza*-derived interventions significantly improved trabecular thickness (MD = 11.70, 95% CI = 7.95 to 15.45, p < 0.000001). Figure 5 illustrates the effect of *S. miltiorrhiza*-derived interventions on bone volume fraction and trabecular separation. Twelve studies reported improvements in bone volume fraction (SMD = 2.47, 95% CI = 1.64 to 3.30, p < 0.000001), while 14 studies showed its impact on trabecular separation (MD = −100.07, 95% CI = −129.23 to −70.90, p < 0.000001).

### Biomechanical parameters of bone

**FIGURE 6:**
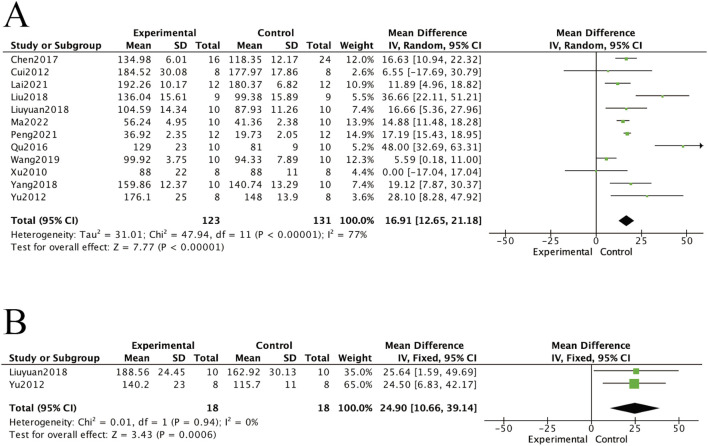
Forest plots of the effects of *Salvia miltiorrhiza*-derived interventions on bone biomechanical strength in osteoporosis animal models. **(A)** Maximum load. **(B)** Ultimate stress.

**FIGURE 7:**
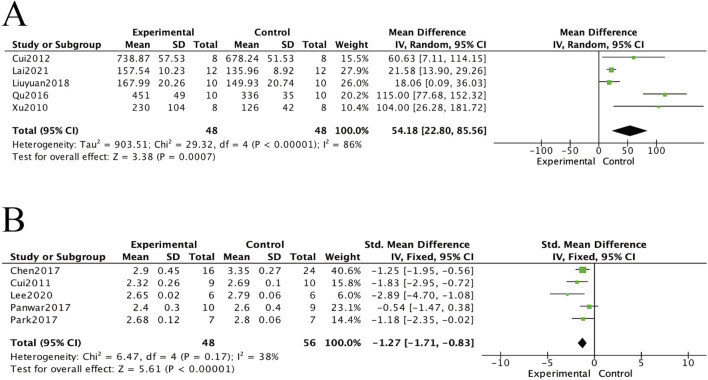
Forest plots of the effects of *Salvia miltiorrhiza*-derived interventions on bone elastic modulus and structural model index in osteoporosis animal models **(A)**. Elastic modulus. **(B)** Structural model index.

The meta-analysis on the effects of *S. miltiorrhiza*-derived interventions on bone biomechanical parameters in OP animal models is shown in Figures 6, 7. Figure 6 shows the effects on maximum load and ultimate stress. Twelve studies summarized the results for maximum load (MD = 16.91, 95% CI = 12.65 to 21.18, p < 0.000001); two studies reported results for ultimate stress (MD = 24.90, 95% CI = 10.66 to 39.14, p < 0.000001). Because only two studies reported ultimate stress, this result should be interpreted cautiously and regarded as exploratory. Figure 7 presents the results for elastic modulus and structural model index (SMI). Five studies reported results for elastic modulus (MD = 54.18, 95% CI = 22.80 to 85.56, p < 0.000001), and five studies reported results for SMI (SMD = −1.27, 95% CI = −1.71 to −0.83, p < 0.000001).

### Biochemical markers of bone metabolism

**FIGURE 8:**
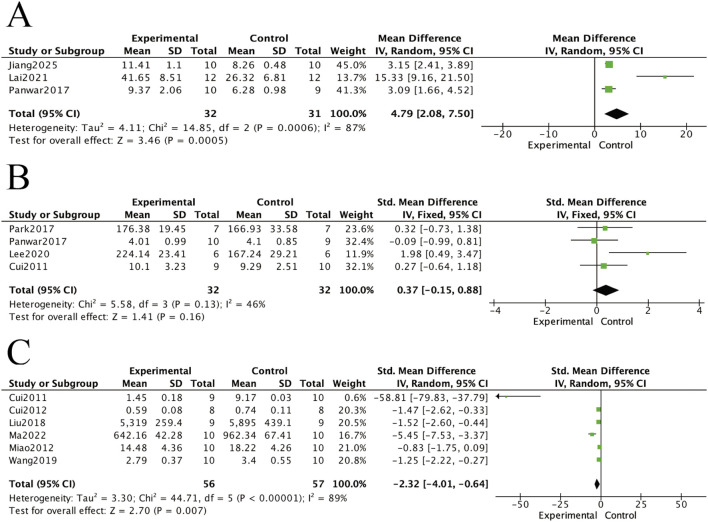
Forest plots of the effects of *Salvia miltiorrhiza*-derived interventions on biochemical markers of bone metabolism in osteoporosis animal models. **(A)** Procollagen type I N-terminal propeptide. **(B)** Estradiol. **(C)** Tartrate-resistant acid phosphatase.

**FIGURE 9:**
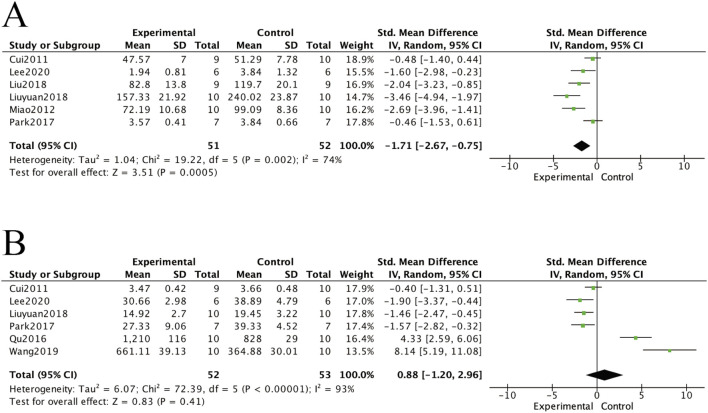
Forest plots of the effects of *Salvia miltiorrhiza*-derived interventions on serum alkaline phosphatase and osteocalcin in osteoporosis animal models. **(A)** Serum alkaline phosphatase. **(B)** Serum osteocalcin.

**FIGURE 10:**
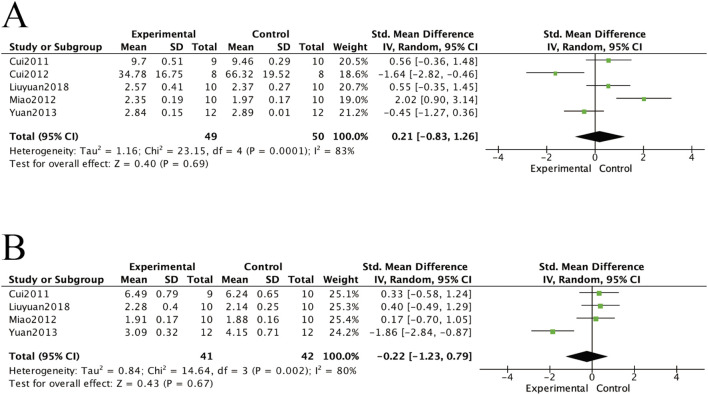
Forest plots of the effects of *Salvia miltiorrhiza*-derived interventions on serum calcium and serum phosphorus in osteoporosis animal models. **(A)** Serum calcium. **(B)** Serum phosphorus.

Figures 8–10 present the meta-analysis results of bone metabolism and bone biochemical markers after intervention with *S. milti*orrhiza-derived interventions in OP animal models. In Figures 3, 8 studies reported the results for PINP (MD = 4.79, 95% CI = 2.08 to 7.50, p = 0.0005), 4 studies provided serum E2 results (SMD = 0.37, 95% CI = −0.15 to 0.88, p = 0.16), and 6 studies reported results for TRACP (SMD = −2.32, 95% CI = −4.01 to −0.64, p = 0.007). Because only 3 studies contributed to the PINP analysis, this finding should be interpreted cautiously and regarded as exploratory. Figure 9 summarizes the meta-analysis results for ALP and OC. Six studies reported the effects of *S. miltiorrhiza* intervention and control intervention on ALP (SMD = −1.71, 95% CI = −2.67 to −0.75, p = 0.0005), and 6 studies provided the meta-analysis results for OC (SMD = 0.88, 95% CI = −1.20 to 2.96, p = 0.41), with no statistical significance. Figure 10 presents the results of the meta-analysis for serum calcium and phosphorus. Five studies reported results for serum calcium (SMD = 0.21, 95% CI = −0.83 to 1.26, p = 0.69), and four studies contributed to the meta-analysis for serum phosphorus (SMD = −0.22, 95% CI = −1.23 to 0.79, p = 0.67), with both showing no statistical significance.

### Sensitivity analysis results and publication bias

**FIGURE 11:**
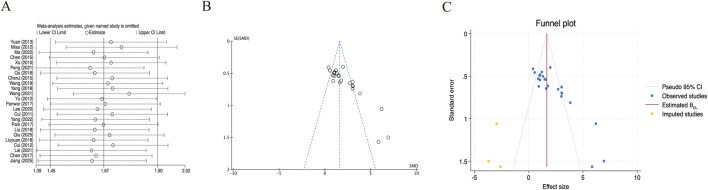
Sensitivity and publication bias analyses. **(A)** Leave-one-out sensitivity analysis. **(B)** Funnel plot for assessment of publication bias. **(C)** Trim-and-fill analysis, with three imputed studies added to account for funnel plot asymmetry.

According to the results of the sensitivity analysis (Figure 11A), after excluding each study individually, the combined effect estimates remained between 1.45 and 1.90, and none of the estimates or their 95% confidence intervals crossed the line of no effect. This indicates that the overall effect direction remained unchanged regardless of which study was excluded, and the conclusion remained consistent and stable. 1n the figure, each study point and its corresponding confidence interval show that most studies had minimal impact on the combined effect, with no single study excessively influencing the overall result. Therefore, the results of this meta-analysis demonstrate good stability and reliability. After excluding studies judged to be at high risk of bias, 9 studies remained, and the pooled effect size remained statistically significant (SMD = 2.42, 95% CI 1.51 to 3.32; Z = 5.23, P < 0.00001), indicating that the main conclusion was not materially changed. However, residual heterogeneity remained relatively high (I^2^ = 75%), and the results should therefore be interpreted cautiously. Visual inspection of the funnel plot (Figure 11B) suggested asymmetry, and Egger’s regression test was statistically significant (P < 0.05), indicating potential small-study effects and publication bias. In the trim-and-fill analysis (Figure 11C), 3 studies were imputed on the left side of the funnel plot. After adjustment, the pooled effect size decreased from 1.951 (95% CI: 1.481–2.421) to 1.671 (95% CI: 1.145–2.197), while the overall effect remained statistically significant. These findings suggest that the relatively large pooled effect size may have been influenced, at least in part, by methodological bias or small-study effects; however, because the direction and statistical significance of the effect remained unchanged across the sensitivity, high-risk-of-bias exclusion, and trim-and-fill analyses, the main conclusion of this meta-analysis appears to be robust, although the magnitude of the pooled effect should be interpreted cautiously.

## Discussion

This study employed a systematic review and meta-analysis to evaluate the therapeutic effects of *S. miltiorrhiza*-derived interventions in different OP animal models. The results showed that *S. miltiorrhiza*-derived interventions significantly improved BMD, bone tissue morphology (such as trabecular number and trabecular thickness), and bone biomechanical parameters (such as maximum load and ultimate stress). These findings provide preclinical evidence supporting the potential bone-protective effects of *S. miltiorrhiza*-derived interventions in experimental OP models. Specifically, *S. miltiorrhiza*-derived interventions significantly improved BMD (SMD = 1.95, 95% CI = 1.48 to 2.42, p < 0.000001), improved trabecular structure, increased trabecular number and thickness, and enhanced bone biomechanical properties. However, some biomechanical outcomes, particularly ultimate stress, were based on a very limited number of studies and should therefore be interpreted cautiously as exploratory findings. Furthermore, the observed changes in bone metabolism markers, including increased PINP and reduced TRACP and ALP, may provide preliminary biomarker signals supporting the bone-protective effects of *S. miltiorrhiza*-derived interventions. However, outcomes based on a small number of studies, especially PINP, should also be interpreted cautiously. Importantly, compared with previous evidence syntheses centered on single constituents or qualitative summaries, the present study provides a broader quantitative integration of *S. miltiorrhiza*-derived interventions across multiple osteoporosis models and identifies efficacy patterns related to model type, active substance category, dose, and intervention duration.

*Salvia miltiorrhiza*-derived interventions significantly improved BMD (SMD = 1.95, 95% CI = 1.48 to 2.42, p < 0.000001), suggesting a potential pharmacological effect against bone loss in animal models of OP. BMD is a key indicator of bone health, directly affecting bone strength and fracture risk (Xia et al., 2022). By improving BMD, *S. miltiorrhiza*-derived interventions help enhance bone mechanical strength, thereby reducing the incidence of fractures. Clinically, *S. miltiorrhiza* injection combined with calcium or calcium/vitamin D treatment for osteoporotic fractures demonstrated greater efficacy than calcium or calcium/vitamin D alone. This combination effectively reduces inflammation, regulates bone metabolism, and improves fracture healing, while maintaining good safety (Li et al., 2024). The active components in *S. miltiorrhiza*-derived interventions, such as tanshinones and salvianolic acids, have been reported to possess antioxidant and anti-inflammatory properties. These properties may contribute to reduced bone resorption and enhanced osteogenesis (Yang et al., 2025; Chen et al., 2025). Tanshinone has been reported to promote the formation and mineralization of osteoblasts and to inhibit excessive bone resorption under disease conditions (Sudha et al., 2025). Furthermore, Tanshinone IIA has been reported to significantly reverse dexamethasone-induced inhibition of osteogenesis and apoptosis in bone marrow mesenchymal stem cells (BMSCs), potentially through activation of the ERK1/2-CREB signaling pathway (Li X. et al., 2025). Tanshinone has also been reported to attenuate OVX-induced OP and BMSCs senescence by upregulating phosphoglycerate dehydrogenase, thereby helping preserve bone structure and stem cell characteristics in OVX rats (Wang L. et al., 2019). Salvianolic acid C (SAC) may promote osteogenic differentiation of OVX rat BMSCs by activating the AMPK/SIRT1 signaling pathway (Liu et al., 2023). Additionally, salvianolic acid A (SAA) has been reported to inhibit osteoclastogenesis and bone resorption *in vitro* and in OVX-induced osteoporotic mice by reducing reactive oxygen species generation, activating the Nrf2/HO-1 pathway, and inhibiting the NF-κB and MAPK signaling pathways (Qiu et al., 2025). Salvianolic acid A (SAA) has also been reported to promote osteogenic differentiation by inhibiting the Notch1 signaling pathway and to suppress osteoclast differentiation by inhibiting the NF-κB pathway (Cao et al., 2025). However, these mechanistic pathways were not quantitatively analyzed in the present meta-analysis and should therefore be regarded as hypothesis-generating interpretations rather than evidence-based conclusions of this study.

*Salvia miltiorrhiza*-derived interventions significantly increased trabecular number (MD = 0.77, 95% CI = 0.51 to 1.04, p < 0.000001), improved trabecular thickness (MD = 11.70, 95% CI = 7.95 to 15.45, p < 0.000001), and significantly reduced trabecular separation (MD = −100.07, 95% CI = −129.23 to −70.90, p < 0.000001), indicating its significant impact on improving bone quality. This effect may be related to the activation of osteoblasts, inhibition of osteoclasts, regulation of oxidative stress, and suppression of inflammation by *S. miltiorrhiza*-derived interventions in animals (Kim et al., 2008; Ye et al., 2023; Liu et al., 2021). For example, Tanshinone IIA has been reported to markedly promote the differentiation of osteoblasts and the deposition of bone matrix, concurrently suppressing both the generation and function of osteoclasts, thereby suggesting a pronounced capacity to counteract OP. Such skeletal protection may be related, at least in part, to the compound’s ability to block NF-κB signaling and reduce the expression of pro-inflammatory cytokines that promote bone breakdown (Hu et al., 2025). Additionally, salvianolic acid may contribute to improved bone mass and trabecular microarchitecture, potentially through modulation of the RANKL/RANK/OPG pathway and suppression of inflammatory mediators such as TNF-α (Gao et al., 2021). Nevertheless, these proposed pathways were not quantitatively assessed in the present meta-analysis and should be interpreted as mechanistic hypotheses rather than direct conclusions of this study.

*Salvia miltiorrhiza*-derived interventions significantly improved bone metabolic markers, specifically by significantly increasing PINP levels (MD = 4.79, 95% CI = 2.08 to 7.50, p = 0.0005), suggesting promotion of osteoblast function and bone formation; *S. miltiorrhiza* slightly increased serum E2 levels (SMD = 0.37, 95% CI = −0.15 to 0.88, p = 0.16), but this effect did not reach statistical significance, indicating that its influence on estrogen-related metabolism in OP may be limited; *S. miltiorrhiza* significantly reduced TRACP levels (SMD = −2.32, 95% CI = −4.01 to −0.64, p = 0.007), indicating its ability to inhibit osteoclast activity and reduce bone resorption; At the same time, it significantly reduced ALP levels (SMD = −1.71, 95% CI = −2.67 to −0.75, p = 0.0005), which may reflect modulation of bone turnover. *Salvia miltiorrhiza* regulates key bone metabolic markers, balancing osteogenesis and osteoclastogenesis. By increasing PINP levels, *S. miltiorrhiza* promotes osteogenesis. Additionally, by lowering TRACP and ALP levels, it effectively inhibits excessive bone resorption and helps restore bone metabolic balance. Studies have shown that salvianolic acid B (Sal B) significantly promotes osteoblast activity and ALP expression by upregulating the expression of osteogenesis and differentiation-related genes (such as Runx2, OC), providing protective effects and alleviating the suppressive effects of prednisolone acetate on osteoblasts (Qiao et al., 2019).

Because the included interventions comprised both crude extracts and chemically distinct bioactive metabolites derived from *S. miltiorrhiza*, the overall pooled estimate should be interpreted cautiously as a class-level summary rather than evidence that all included interventions are pharmacologically equivalent. To improve interpretability, we performed subgroup analysis by phytochemical class, which showed generally favorable effects across aqueous extract, ethanolic extract, tanshinone-class, and salvianolic acid-class interventions, although the magnitude of effect varied among subgroups. This suggests that the anti-osteoporotic effects may be shared at the botanical source level, while still differing quantitatively according to chemical composition. Dose interpretation in this meta-analysis also requires caution. Although the reported doses ranged from 5 mg/kg/day to 15 g/kg/day, these values were derived from interventions with substantially different compositions, including crude extracts and isolated bioactive metabolites. Therefore, the nominal dose values were not directly comparable across studies in a pharmacological sense. In particular, the highest doses were mainly reported in crude extract studies, whereas purified constituents were generally administered at much lower mg/kg doses. Accordingly, the dose-based subgroup analysis should be regarded as exploratory rather than as evidence of a true dose-response relationship. The subgroup analysis of this study further revealed that multiple factors may influence the efficacy of *S. miltiorrhiza* in the treatment of OP, including the type of OP model, animal species, phytochemical class of intervention, intervention dose, and duration.

In particular, the efficacy of *S. miltiorrhiza*-derived interventions was particularly notable in the OVX-induced OP model. The OVX model is widely regarded as a classic animal model for postmenopausal OP, characterized by rapid bone loss induced by decreased estrogen levels, with the bone loss worsening over time (Kharode et al., 2008). *Salvia miltiorrhiza*-derived interventions exhibited enhanced bone-protective effects in this model, with significant effects when the intervention duration was less than 12 weeks. This suggests that its mechanism of action extends beyond promoting osteogenesis, potentially also involving anti-bone-loss effects, thus achieving a dual regulation of bone metabolism. The timing of OP treatment plays a crucial role in its efficacy. Network meta-analysis (NMA) indicated that the efficacy of OP treatment varies significantly depending on treatment duration (e.g., 12, 24, or 36 months), with treatment outcomes closely related to the treatment regimen (such as romosozumab (ROMO), teriparatide (TPTD), and abaloparatide (ABL)) and BMD results (Willems et al., 2022). This finding underscores the critical role of treatment timing and regimen in the management of OP. Additionally, while subgroup analysis suggested a more robust pooled effect in SD rats, this may simply reflect a statistical artifact due to the disproportionately high number of studies utilizing this specific strain in OP research, rather than a true strain-specific biological sensitivity. Notably, tanshinone class interventions demonstrated higher efficacy in the subgroup analysis, likely due to their comprehensive pharmacological effects. Tanshinones are terpenoid compounds with strong lipophilicity, encompassing anti-inflammatory, antioxidant, and osteogenesis-promoting properties (Xiao et al., 2020). It is noteworthy that the potential clinical translation of tanshinone derivatives is still limited by various pharmacokinetic barriers, including poor water solubility, low oral absorption, limited bioavailability, and rapid clearance. However, nanotechnology-based delivery systems and optimized formulation techniques may help address these pharmacokinetic barriers (Hu et al., 2025).

## Limitation

Despite the promising results demonstrated in this meta-analysis, several limitations should be considered. First, while the inclusion of studies across different animal models provides a comprehensive assessment, the variations in animal species, models, and the methods of intervention may introduce heterogeneity that could affect the generalizability of the results. For example, the OVX model is widely used for postmenopausal OP, but other models, such as glucocorticoid-induced and diabetes-induced OP, were also included, which might respond differently to treatment. Moreover, the intervention duration and dosage varied significantly across studies, which may impact the overall efficacy of *S. miltiorrhiza*-derived interventions. Moreover, the broad dose range reported across studies should not be interpreted as directly clinically translatable, because crude extracts and purified constituents differ substantially in composition, potency, and likely pharmacokinetic behavior. In addition, several secondary outcomes were based on a very limited number of studies, such as ultimate stress (n = 2) and PINP (n = 3), which increase the uncertainty of these pooled estimates and raise the possibility of false-positive findings. Therefore, these results should be considered exploratory and require confirmation in future studies. In addition, the included interventions encompassed multiple phytochemical classes and both extracts and isolated constituents. Therefore, the overall pooled effect should not be interpreted as evidence of pharmacological interchangeability among all *S. miltiorrhiza*-derived interventions. Because the number of studies within many individual compound categories was limited, fully compound-specific meta-analyses were not feasible and should be addressed in future studies. Second, the quality of the included studies could be a concern. Despite following rigorous inclusion criteria, most studies did not report detailed information about random sequence generation, allocation concealment, or blinding of outcome assessors. This may lead to potential bias, affecting the reliability of the findings. Many studies were rated as having unclear rather than confirmed high risk in some domains because key methodological details were insufficiently reported. Therefore, the available evidence should be interpreted cautiously as low-certainty preclinical evidence. In addition, the relatively large pooled effect size for BMD may have been influenced, at least in part, by methodological bias or small-study effects. Although sensitivity analysis excluding high-risk-of-bias studies and trim-and-fill analysis did not materially change the overall direction or statistical significance of the main finding, the adjusted effect size was attenuated, suggesting that the magnitude of the pooled effect should be interpreted cautiously. Furthermore, the studies predominantly used animal models, and the findings may not fully translate to clinical human applications, as animal models do not always replicate human responses accurately. In particular, commonly used rodent models such as ovariectomized (OVX) rats reproduce only selected aspects of human osteoporosis and cannot fully capture the complexity of human disease progression, treatment response, or interspecies pharmacokinetic differences associated with botanical drug interventions. Finally, although *S. miltiorrhiza* has shown significant efficacy, the pharmacokinetic limitations of its active compounds, such as poor water solubility and low bioavailability, remain challenges for clinical use. Although nanotechnology-based delivery systems show promise in overcoming these barriers, more research is needed to optimize these formulations for effective clinical translation.

## Data Availability

The original contributions presented in the study are included in the article/Supplementary Material, further inquiries can be directed to the corresponding author.
