# Supplementary material for: Pharmacological effects of Salvia miltiorrhiza-derived interventions on osteoporosis in animal models: a systematic review and meta-analysis
Source: Front Pharmacol. 2026 May 26;17:1740974. doi: 10.3389/fphar.2026.1740974 (PMC13247436; doi:10.3389/fphar.2026.1740974)
Supplement: Supplementary file 1 [file Supplementaryfile1.docx]

**Pubmed**

(("Salvia miltiorrhiza"[Title/Abstract] OR "Danshen"[Title/Abstract] OR "Tanshinone"[Title/Abstract]) AND ("Osteoporosis"[MeSH Terms] OR "osteoporosis"[Title/Abstract] OR "bone loss"[Title/Abstract] OR "bone resorption"[Title/Abstract]) AND ("Rats"[MeSH Terms] OR "Mice"[MeSH Terms] OR rat[Title/Abstract] OR rats[Title/Abstract] OR mouse[Title/Abstract] OR mice[Title/Abstract] OR rodent[Title/Abstract] OR rodents[Title/Abstract]))

**Web of Science**

TS=("Salvia miltiorrhiza" OR Danshen OR Tanshinone) AND TS=(osteoporosis OR "bone loss" OR "bone resorption") AND TS=(rat OR rats OR mouse OR mice OR rodent OR rodents)

**Embase**

(('salvia miltiorrhiza'/exp OR 'danshen':ti,ab,kw OR 'tanshinone':ti,ab,kw) AND ('osteoporosis'/exp OR 'osteoporosis':ti,ab,kw OR 'bone loss':ti,ab,kw OR 'bone resorption':ti,ab,kw) AND ('rat'/exp OR 'mouse'/exp OR 'rat':ti,ab,kw OR 'rats':ti,ab,kw OR 'mouse':ti,ab,kw OR 'mice':ti,ab,kw OR 'rodent':ti,ab,kw OR 'rodents':ti,ab,kw))

**FMRS**

(("Salvia miltiorrhiza" OR "Danshen" OR "Tanshinone" OR "Danshen root" OR "Salvia root") AND ("Osteoporosis" OR "Bone loss" OR "Bone resorption" OR "Bone density loss" OR "Low bone mass" OR "Postmenopausal osteoporosis" OR "Osteopenia") AND ("Rat" OR "Rats" OR "Mouse" OR "Mice" OR "Rodent" OR "Rodents" OR "Laboratory rat" OR "Laboratory mouse" OR "Animal model"))

**Scopus**

(TITLE-ABS-KEY("Salvia miltiorrhiza" OR "Danshen" OR "Tanshinone" OR "Danshen root" OR "Salvia root")) AND (TITLE-ABS-KEY("Osteoporosis" OR "Bone loss" OR "Bone resorption" OR "Bone density loss" OR "Low bone mass" OR "Postmenopausal osteoporosis" OR "Osteopenia")) AND (TITLE-ABS-KEY("Rat" OR "Rats" OR "Mouse" OR "Mice" OR "Rodent" OR "Rodents" OR "Laboratory rat" OR "Laboratory mouse" OR "Animal model"))
